# From Material to Medicine: Translational Frontiers in Dosage Form Design for Oral Administration

**DOI:** 10.3390/pharmaceutics17121529

**Published:** 2025-11-27

**Authors:** Hend E. Abdelhakim, Atheer Awad

**Affiliations:** 1 Global Business School for Health, University College London, Marshgate, London E20 2AE, UK; 2 Department of Clinical, Pharmaceutical and Biological Sciences, University of Hertfordshire, Hatfield AL10 9AB, UK

## 1. Introduction

Oral administration remains the most patient-preferred and widely adopted route for medicines, yet it increasingly challenges formulation science to overcome poor solubility, instability, and acceptability barriers while supporting dose flexibility and manufacturability. The Special Issue ‘Dosage Form Design for Oral Administration’ brings together five original research papers illustrating how advanced materials, automated manufacturing, and patient-centred design can converge to create next-generation oral dosage forms. Collectively, they reflect a shift from isolated formulation optimisation to integrated, translational, and data-enabled approaches capable of addressing both clinical and system-level needs.

## 2. Overview of Published Work

The Special Issue showcases complementary innovations across enabling technologies, including nanocomplexes, gastro-retentive systems, taste-masking, electrospraying and additive manufacturing.

Tihhonova et al. [1] demonstrated automated extrusion-based deposition of prednisolone gel tablets, highlighting the role of semi-solid 3D printing in personalised drug dosing and veterinary care. By achieving precise weight control and rapid disintegration, the study exemplifies how digital manufacturing can ensure reproducibility while allowing patient-specific tailoring.

Volitaki et al. [2] used electrospraying to load itraconazole into mesoporous silica, maintaining the amorphous state and achieving an order-of-magnitude improvement in dissolution properties. Their work highlights how carrier-based amorphisation offers a scalable route to enhance bioavailability for poorly soluble BCS II/IV drugs and supports the growing emphasis on solvent-lean, continuous processing.

Kim et al. [3] developed an ionic nanocomplex of semaglutide with organometallic phyllosilicate protected by a pH-responsive polymer coating. Improved stability, epithelial transport, and glycaemic control in diabetic rats signal progress toward viable oral delivery of peptides and other biologics through colonic targeting and multi-layered protection.

Siripruekpong et al. [4] formulated in-situ gelling, raft-forming liquids containing curcumin and resveratrol. The approach prolongs gastric residence times, enhances solubility, and sustains release of polyphenolic compounds, demonstrating that familiar, patient-friendly dosage forms can deliver sophisticated control of release and retention.

Kovalenko et al. [5] produced taste-masked warfarin pellets using Kollicoat^®^ Smartseal, achieving negligible salivary release and controlled gastric dissolution. The formulation addresses dose personalisation for drugs with narrow therapeutic indices and reinforces the importance of palatability and adherence in paediatric and geriatric care.

Together, these contributions reveal how smart materials and modern manufacturing converge with patient-centric design to create dosage forms that are effective, acceptable, and potentially scalable. They also demonstrate the translational maturity of current research, where proof-of-concept and early in-vivo validation are achieved, but regulatory, industrial, and health-system integration remain the next frontier.

## 3. Future Perspectives

The next phase of oral dosage form innovation will depend on bridging scientific, digital, behavioural, and translational domains. Artificial Intelligence (AI) and digital twins are beginning to reshape formulation design, enabling rapid prediction of excipient compatibility, dissolution kinetics, and process control. When combined with continuous manufacturing, these tools promise data-driven optimisation and reduced development cycles. Yet AI must be coupled with transparent datasets, explainable models, and regulatory engagement to ensure reproducibility and trustworthiness [1,2].

At the same time, effective translation demands early patient and public involvement (PPI) and behavioural insight. Co-designing dosage forms with patients and caregivers ensures that features such as taste, texture, and ease of administration are integral to product performance [3]. Acceptability research consistently shows that palatability, flexible dosing, and discrete, socially acceptable formats increase adherence and therapeutic success [4].

To progress from Technology Readiness Level (TRL) 5 toward clinical adoption (TRL 6–9), interdisciplinary collaboration is essential. Academia can de-risk early innovation; industry can ensure scalability and quality; and health systems can define value through outcomes and access. Within the UK, the National Health Services (NHS) long-term plan emphasises digital-first, community-based care and AI-enabled therapeutics [5,6,7]. Oral formulations that align with these priorities, including smart, personalised, remotely monitorable dosage forms, will have the greatest translational potential.

Looking forward, several priorities stand out. First, integrating AI and digital analytics across formulation and manufacturing will be essential to enable predictive, efficient, and sustainable design. Second, embedding PPI alongside behavioural science approaches can strengthen acceptability and real-world adherence. Third, aligning advancements with health system goals, including those outlined in the NHS Plan, which will help ensure that new technologies are not only innovative but also practical and implementable. Finally, mapping TRLs to regulatory and reimbursement pathways will be critical to ensure that innovations can successfully transition from research to industrial scale and health-system adoption.

By combining material science with data science, and technical excellence with human insight, the next generation of oral dosage forms can move from innovation to real-world impact.
